# Digital Transformation in Pathology: A Digital‐First Model for Integrated Diagnostic Systems

**DOI:** 10.1111/pin.70171

**Published:** 2026-09-06

**Authors:** Hiroyuki Kusano, Takayuki Shiomi

**Affiliations:** ^1^ Graduate School of Medicine International University of Health and Welfare Narita Chiba Japan; ^2^ Department of Anatomic Pathology IUHW Narita Hospital Narita Chiba Japan; ^3^ Department of Pathology International University of Health and Welfare, School of Medicine Narita Chiba Japan

## Abstract

Digital pathology has rapidly expanded over the past decade, driven by advances in whole‐slide imaging and increasing demand for remote and data‐integrated diagnostic workflows. However, many implementations remain limited to partial digitization, without fundamentally transforming laboratory and diagnostic processes. In this review, we examine digital transformation in pathology as a systems‐level redesign encompassing workflow optimization, digital infrastructure, and networked diagnostic services. Using the implementation at International University of Health and Welfare (IUHW) Narita Hospital as an illustrative example, we describe how a digital‐first approach can be integrated into routine clinical practice. Key elements include continuous laboratory workflows aligned with slide digitization, comprehensive digital infrastructure enabling seamless access to diagnostic data, and networked systems that support multi‐institutional collaboration and subspecialty consultation. We further discuss how digital pathology extends beyond clinical diagnosis to support education and research, transforming traditional slide archives into accessible data resources, and outline practical considerations for implementation, including workflow redesign, user adoption, and institutional coordination. Finally, by comparing the IUHW Narita experience with other large‐scale implementations, we discuss generalizable principles for digital transformation in pathology, including workflow‐driven design, data integration, interoperability, and network‐based diagnostic models.

## Graphical abstract

Digital transformation in pathology extends beyond replacing glass slides with digital images. It requires systems‐level redesign integrating laboratory workflow, digital‐first diagnosis, data management, interoperability, and networked diagnostic services. Drawing on the IUHW Narita model and other large‐scale implementations, this review discusses practical principles for developing integrated digital pathology systems across diverse institutional settings.

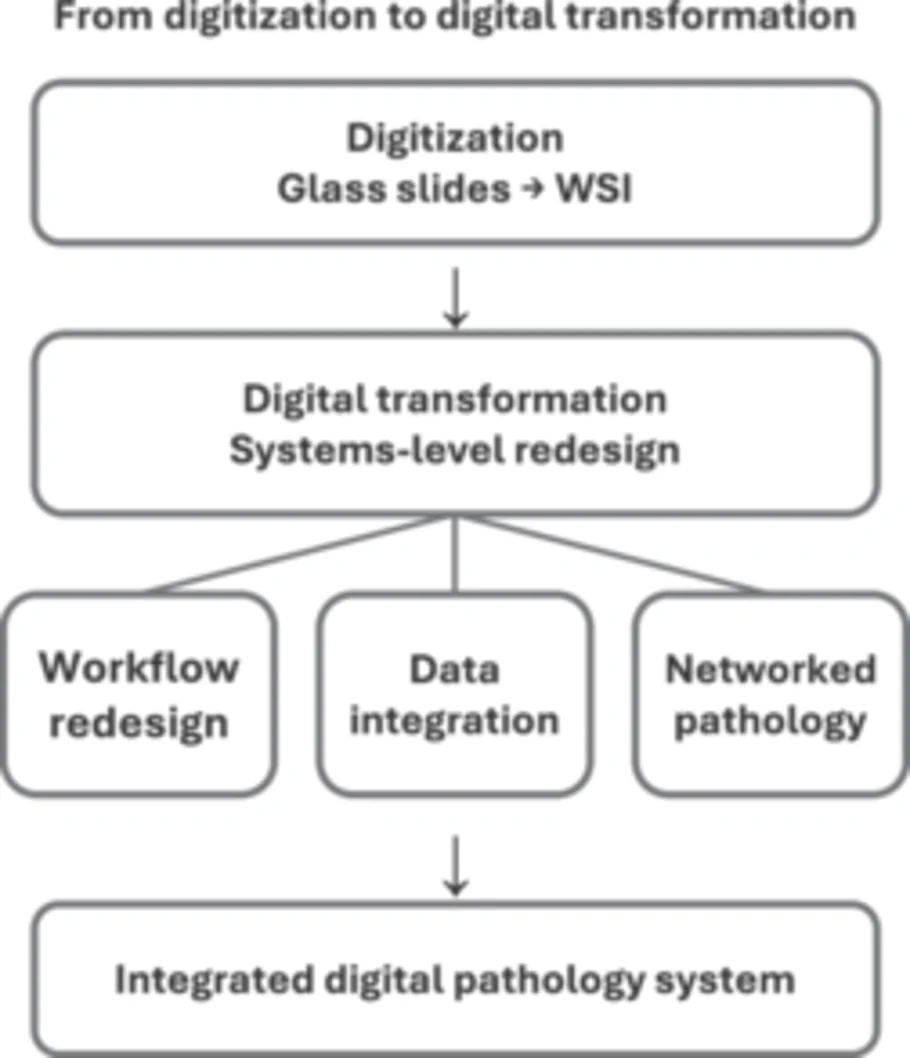

## 1 Introduction

Anatomic pathology has undergone rapid transformation over the past decade. Traditionally, diagnosis relied on analog workflows centered on light microscopy and physical glass slides. However, advances in whole‐slide imaging (WSI) have enabled the broader adoption of digital pathology (DP), supporting remote sign‐out, digital consultation, and distributed diagnostic workflows [1, 2]. The COVID‐19 pandemic further accelerated this trend in many institutions, although the design and implementation of digital‐first systems in some settings predated the pandemic.

Despite this progress, an important distinction exists between digitization and digital transformation. Digitization refers to the conversion of glass slides into digital images, whereas digital transformation involves the redesign of workflows, information systems, and diagnostic processes to create new clinical and organizational value. In many institutions, DP implementation remains limited to partial digitization—such as selective slide scanning or conference use—without fundamentally altering diagnostic workflows [3]. These fragmented approaches often fail to realize the full potential of digital pathology. In such settings, digital pathology risks being reduced to a superficial technological upgrade rather than a true transformation of diagnostic practice. In this context, system‐level redesign—rather than technology‐first implementation—is essential for meaningful transformation.

Radiology provides a useful precedent. The transition from film‐based imaging to picture archiving and communication systems (PACS) reshaped not only image storage and viewing but also clinical workflows and interdepartmental collaboration. A comparable transformation is now emerging in pathology, although its implementation remains heterogeneous across institutions [3, 4].

**Figure 1:**
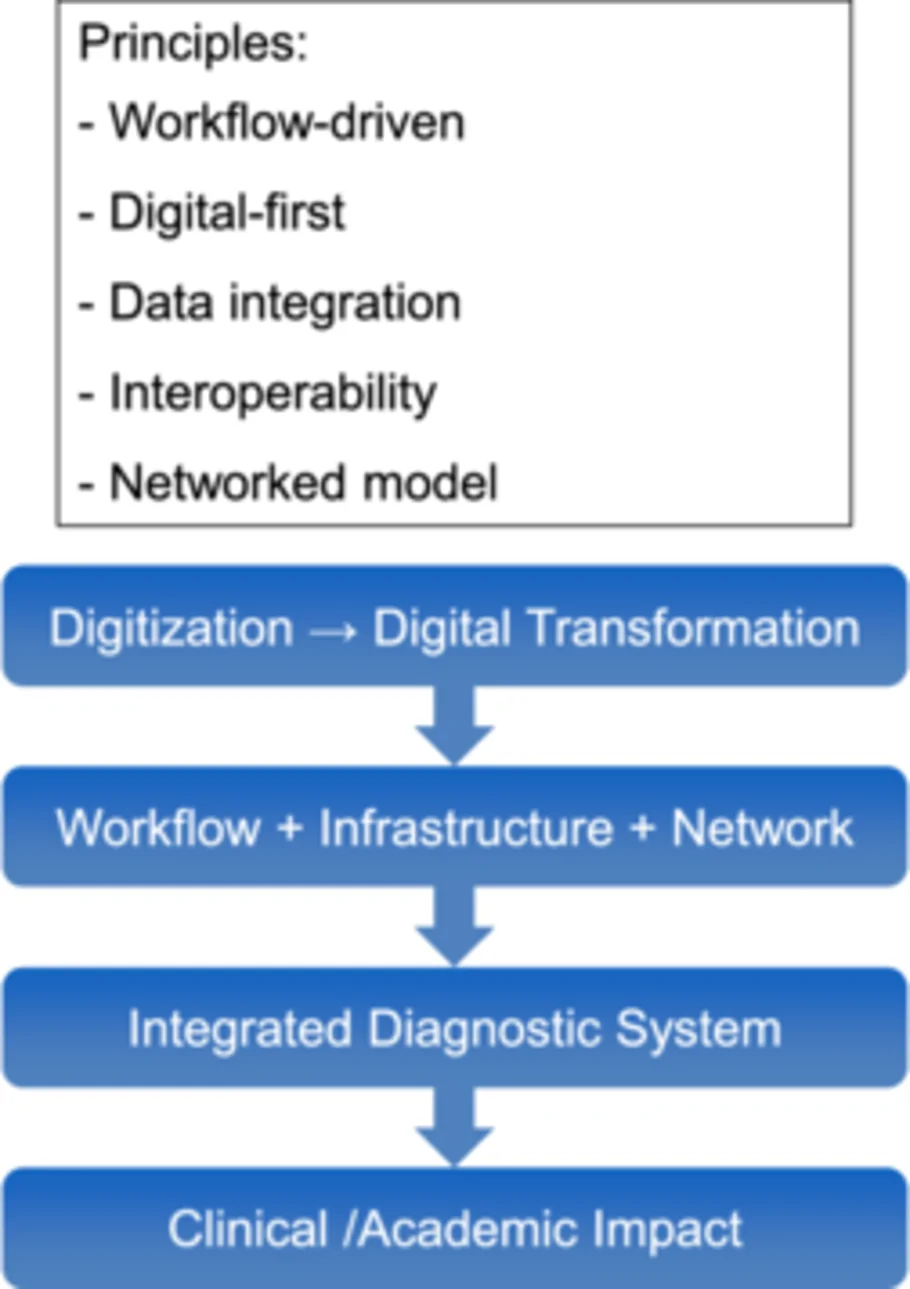
Conceptual framework of digital transformation in pathology. Digital transformation in pathology extends beyond slide digitization to encompass workflow redesign, digital infrastructure, and networked diagnostic systems. These components enable integrated diagnostic environments and broader clinical and academic impact. Core principles include workflow‐driven design, digital‐first diagnosis, data integration, interoperability, and network‐based practice.

Digital transformation in pathology can therefore be understood as a systems‐level redesign encompassing laboratory workflows, digital infrastructure, and networked diagnostic services. This conceptual framework is summarized in Figure 1. The experience at International University of Health and Welfare (IUHW) Narita Hospital provides a representative example of this approach. Established in 2020 without legacy infrastructure, the institution implemented a digital‐first model integrating high‐throughput slide digitization, workflow optimization, and multi‐institutional collaboration.

This review describes the conceptual framework and practical implementation of digital transformation in pathology, using the IUHW Narita model as an illustrative case. It further discusses how such systems enable new forms of diagnostic collaboration, education, and research, and considers their implications for the future of academic pathology. Although many published digital pathology reports focus on scanner deployment, image management, or validation for primary diagnosis, the IUHW Narita model was planned as an integrated system from inception. Pre‐analytical processes were redesigned to support continuous‐flow specimen processing aligned with scanning capacity, comprehensive digitization was adopted as the default diagnostic mode, and domestic as well as international networked pathology services were incorporated into the core system architecture [4, 5, 6, 7]. Although none of these components is unique to IUHW Narita, they were incorporated together from the outset across pre‐analytical workflow, primary digital diagnosis, and domestic and international diagnostic networks. The distinguishing feature of the IUHW Narita model is therefore not any individual technology, but the integration of these components into a digital‐first pathology service.

### 1.1 From Digitization to Digital Transformation in Pathology

Digital pathology is often introduced through the installation of slide scanners and image management systems. While these technologies are essential, they represent only the first step toward meaningful transformation [5]. In many institutions, digital pathology is implemented as a direct replacement for the microscope, allowing pathologists to view digital slides on computer monitors. Although this approach offers several advantages—including remote access and easier image sharing—it does not fundamentally alter the underlying diagnostic workflow.

True digital transformation requires a broader rethinking of how pathology services operate. Instead of focusing solely on image acquisition, digital transformation integrates digital technologies across the entire diagnostic process, including specimen processing, slide production, image management, data storage, diagnostic collaboration, and integration with molecular and clinical data [2, 5]. In this framework, the digital slide is not simply a replacement for glass slides but becomes a data object embedded within a larger clinical and research ecosystem.

Several structural challenges explain why many digital pathology initiatives stall at the stage of partial digitization [3, 5]. Pathology workflows are complex and highly dependent on pre‐analytical laboratory processes. If specimen processing, slide production, and quality control remain optimized for traditional microscope‐based workflows, digital scanners may become bottlenecks rather than efficiency tools. Similarly, without appropriate storage architecture, network bandwidth, and image management systems, large‐scale WSI archives can become difficult to manage and expensive to maintain.

For these reasons, the successful implementation of digital pathology requires coordinated redesign across multiple domains. Pre‐analytical laboratory processes must be optimized to provide a continuous stream of high‐quality slides suitable for scanning. Information systems must enable seamless integration between laboratory workflows, digital images, and clinical data. Finally, diagnostic workflows must evolve to take advantage of the collaborative and data‐driven capabilities enabled by digital platforms [2, 5].

At IUHW Narita Hospital, digital transformation was approached as a systems‐level redesign rather than a simple technology upgrade. The implementation focused on three interconnected goals: optimizing laboratory workflows to support large‐scale slide digitization, establishing a digital‐first diagnostic environment, and developing a networked pathology service model capable of supporting both local clinical care and multi‐institutional collaboration.

In this context, digital transformation should be understood not as a single endpoint, but as a continuum of organizational change. Institutions may progress through stages of adoption, beginning with limited digitization and advancing toward fully integrated, data‐driven diagnostic environments [2, 5]. Recognizing this continuum is important for setting realistic expectations and guiding implementation strategies.

### 1.2 Workflow Redesign as the Foundation of Digital Pathology

A major barrier to digital pathology implementation is the mismatch between conventional laboratory workflows and the requirements of large‐scale slide digitization [5, 7]. Traditional pathology laboratories are typically organized around batch‐based processing, in which specimens are handled in discrete groups. When applied to digital environments, such workflows can create inefficiencies, with periods of scanner inactivity followed by bottlenecks when large numbers of slides are produced simultaneously.

**Figure 2:**
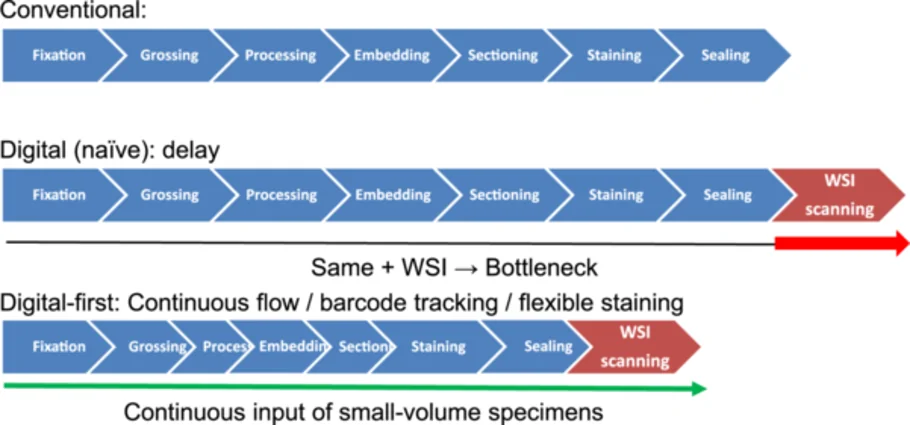
Workflow redesign for digital‐first pathology. Comparison of conventional, naive digital, and digital‐first workflows in pre‐diagnostic laboratory processes. Conventional workflows are batch‐based. In naive digital workflows, the addition of whole‐slide imaging (WSI) introduces delays without fundamental redesign. In contrast, digital‐first workflows incorporate continuous input of small‐volume specimens, barcode tracking, and flexible staining, with the aim of enabling more continuous processing and reducing workflow bottlenecks.

Effective digital transformation therefore requires a shift from batch‐based to continuous‐flow processing. In a continuous workflow, smaller volumes of specimens are processed more frequently, ensuring a steady input of slides into the scanning pipeline and improving overall system efficiency. This principle applies not only to tissue processing but also to downstream steps such as embedding, sectioning, and staining. The relationship between conventional, naive digital, and digital‐first pre‐diagnostic workflows is illustrated in Figure 2.

The implementation at IUHW Narita Hospital illustrates this approach. The laboratory adopted smaller‐capacity processors to enable frequent specimen loading throughout the day, thereby reducing end‐of‐day congestion. A similar strategy was applied to immunohistochemical staining, where flexible workflows allow additional slides to be incorporated into ongoing runs without waiting for large batches. These adjustments are intended to reduce workflow delays, particularly for cases requiring additional diagnostic studies [5, 7].

In addition to workflow continuity, traceability and process integration are essential components of digital transformation [5, 7]. Barcode‐based tracking systems can monitor specimens throughout the laboratory process, reducing identification errors and enabling real‐time status visibility. Integration with laboratory information systems further allows rapid communication of additional test orders, minimizing delays between diagnostic decision‐making and laboratory execution.

These observations highlight a key principle: digital pathology fails when it is implemented at the level of image acquisition alone [5, 7]. Without aligning pre‐analytical processes with the demands of digital workflows, the benefits of whole‐slide imaging cannot be fully realized.

### 1.3 Building a Digital‐First Diagnostic Infrastructure

A digital‐first pathology system requires the integration of whole‐slide imaging, scalable data storage, and interoperable information systems [5, 8]. While individual components such as slide scanners are widely available, their impact depends on how they are incorporated into a unified diagnostic environment.

A central element of digital infrastructure is the adoption of comprehensive slide digitization. Rather than selectively scanning cases, digital‐first systems aim to incorporate whole‐slide imaging into routine workflows, allowing digital images to function as the primary medium for diagnosis. This approach eliminates the need for retrospective scanning and enables consistent access to diagnostic material for clinical, educational, and research purposes.

The implementation at IUHW Narita Hospital reflects this model, with routine digitization of histopathology slides at diagnostic resolution. In such systems, digital slides are integrated with laboratory and clinical information, allowing pathologists to access multiple data sources—including prior cases and ancillary studies—within a single interface. This integration supports more efficient and context‐rich diagnostic workflows.

The transition to digital pathology also requires a reconfiguration of the diagnostic workspace. Digital workstations equipped with high‐resolution displays allow simultaneous visualization of multiple slides and associated clinical data. These environments facilitate rapid navigation across magnifications and enable remote consultation without the need for physical slide transfer, supporting more flexible and collaborative diagnostic practices.

Data management represents a major consideration in digital pathology systems. Whole‐slide images generate large data volumes, necessitating scalable storage solutions. A tiered storage strategy—combining high‐performance storage for active cases with cost‐efficient archival systems—provides a practical approach to balancing performance and long‐term data retention [5, 8]. Automated retrieval of prior cases further enhances usability by integrating historical material into routine diagnostic workflows.

In practice, the scale of digital pathology data continues to expand as institutions move toward comprehensive slide digitization. This trend underscores the importance of designing storage systems that can accommodate long‐term growth while maintaining consistent performance for routine diagnostic use.

At IUHW Narita Hospital, storage and access were considered part of the diagnostic workflow rather than purely technical issues. Recent and active cases require rapid access during sign‐out, whereas older cases are primarily needed for comparison, education, research, or medico‐legal retention. This distinction supports the use of tiered storage, in which high‐performance storage is reserved for active diagnostic material and lower‐cost storage is used for long‐term archives. The practical goal is not simply to store large numbers of whole‐slide images, but to ensure that clinically relevant prior material can be retrieved without disrupting routine diagnostic work.

Interoperability is another critical requirement. Standardized formats such as DICOM reduce dependence on proprietary solutions and support long‐term system flexibility. In radiology, DICOM provides a common format for image storage, metadata, and communication, enabling images from different devices and vendors to be exchanged within unified PACS environments. In pathology, DICOM has been extended to whole‐slide imaging through Supplement 145, but many current systems still rely on proprietary formats, which complicates long‐term data portability and multi‐vendor integration. Moving toward DICOM‐compliant workflows can facilitate linkage between image data, laboratory information, and clinical records, while also supporting computational applications across diverse image sources. At IUHW Narita Hospital, interoperability was treated as a central design requirement, and DICOM‐compatible storage and exchange are prioritized where practical to preserve long‐term flexibility. Interoperable systems also facilitate integration with other medical data sources, including radiologic imaging and clinical records [5, 8].

Finally, digital infrastructure extends beyond individual institutions when supported by network connectivity. Secure network environments enable remote access to digital slides and support multi‐institutional collaboration. In such settings, digital pathology systems function not only as local diagnostic tools but also as components of broader, distributed healthcare networks.

### 1.4 Networked Pathology Services: From Individual Laboratories to Virtual Departments

Digital pathology enables the reorganization of diagnostic services beyond the boundaries of individual laboratories. In conventional workflows, consultation depends on the physical transfer of glass slides, introducing delays and logistical constraints. In contrast, whole‐slide imaging allows cases to be shared instantly within a digital environment, shifting the focus from physical location to diagnostic expertise [4, 9].

This capability supports the development of networked pathology systems in which multiple institutions collaborate within a unified digital framework. In such models, geographically distributed laboratories function as components of a virtual anatomic pathology department, allowing pathologists to access and review cases across institutional boundaries through shared digital platforms [4, 9].

**Figure 3:**
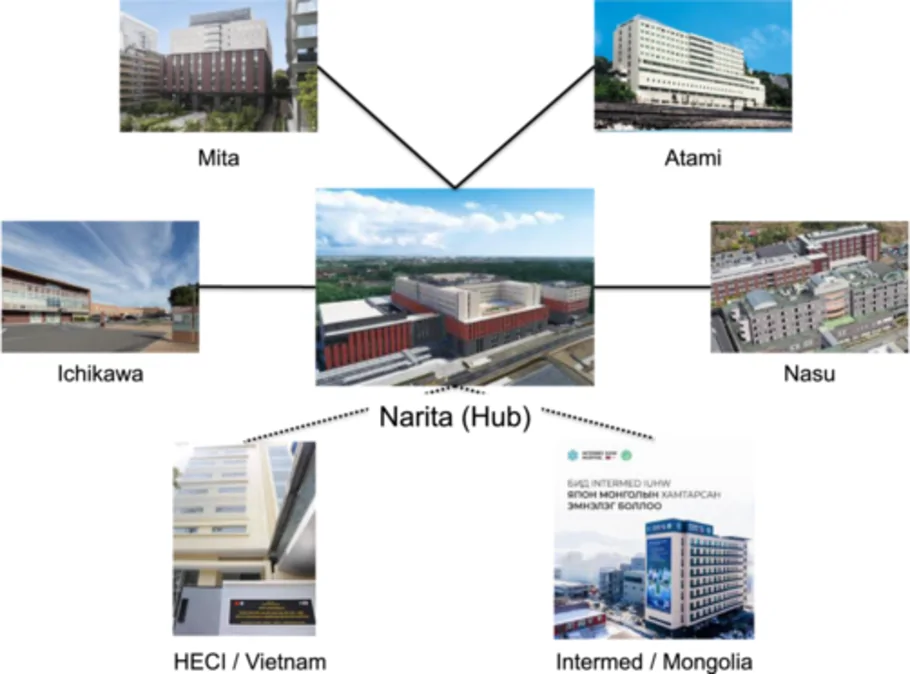
Networked pathology system within the IUHW digital pathology framework. Schematic representation of a multi‐institutional digital pathology network centered at IUHW Narita Hospital. The system connects affiliated hospitals across Japan and includes international collaboration with a site in Vietnam and a planned collaboration with a site in Mongolia. This network supports distributed diagnosis, subspecialty consultation, and shared access to digital pathology resources.

The implementation within the IUHW network provides an illustrative example of this approach at scale. The system connects multiple affiliated hospitals across different regions of Japan, including institutions in Narita and Ichikawa (Chiba), Mita (Tokyo), Atami (Shizuoka), and Nasu (Tochigi), through a shared digital infrastructure. This configuration enables pathologists to access and review cases across geographically distributed sites within a unified diagnostic platform, demonstrating the feasibility of networked pathology in a multi‐hospital setting. More recently, the network has expanded to include international collaboration with HECI in Vietnam and, prospectively, with Intermed Hospital in Mongolia. The overall structure of this networked pathology model is shown in Figure 3. The scale of the IUHW hospital network and pathology workload is summarized in Table 1.

**Table 1:** IUHW hospitals and pathology workload after digital pathology implementation (2025).

Site	Beds	Histopathology specimens	Cytology specimens
IUHW Narita Hospital	642	8616	5171
IUHW Nasu Medical Center	449	6156	4565
IUHW Mita Hospital	291	9787	17139
IUHW Atami Hospital	269	2856	3115
IUHW Ichikawa Medical Center	260	1232	854

*Note:* Values represent post‐implementation workload after stabilization of digital pathology workflows.

A key advantage of networked systems is the ability to support subspecialty consultation. In traditional settings, diagnostic expertise is constrained by local staffing, particularly in smaller hospitals. Digital platforms allow cases to be directed to pathologists with relevant subspecialty expertise regardless of location, enabling more consistent diagnostic quality across institutions [4, 9]. In some configurations, this may include structured secondary review workflows for selected case types, balancing local diagnostic responsibility with centralized expertise.

Networked pathology also facilitates more efficient use of specialized laboratory resources. High‐cost diagnostic procedures, including advanced immunohistochemistry and companion diagnostics, can be centralized at specific sites while maintaining rapid access to results through digital slide sharing. This approach supports quality control, reduces duplication of resources, and improves turnaround times [4].

Beyond institutional networks, digital pathology infrastructure enables international collaboration. When supported by standardized workflows and secure data transfer, whole‐slide images can be interpreted remotely across national boundaries [4, 9]. Such systems support not only diagnostic consultation but also education and capacity building in regions with limited access to subspecialty expertise.

Collectively, these developments illustrate a shift from institution‐centered pathology services to network‐based diagnostic ecosystems. In this model, digital infrastructure enables the distribution of expertise, integration of resources, and expansion of collaborative practice, forming the basis of a virtual pathology department [4, 9].

### 1.5 Digital Pathology as an Educational and Research Platform

Digital pathology extends beyond clinical diagnosis by enabling new approaches to education and research [10, 11]. One immediate advantage is rapid access to historical cases. In conventional workflows, retrieving prior slides can be time‐consuming, whereas digital archives allow pathologists to review previous specimens instantly. This capability supports more comprehensive case evaluation, improves diagnostic safety, and facilitates longitudinal assessment of disease processes.

Digital slide archives also provide a scalable platform for pathology education. Unlike glass slide collections, which are limited by physical access and durability, digital cases can be shared simultaneously among multiple learners. Structured teaching sets can be curated from routine cases, allowing trainees to compare representative examples and develop pattern‐recognition skills. Remote access further enables multi‐institutional teaching activities, supporting consistent training across distributed clinical environments [10, 12].

In addition to education, digital pathology transforms traditional archives into searchable research resources [10, 11]. When linked with diagnostic reports and clinical data, whole‐slide images can be queried to identify cases meeting specific study criteria. This capability simplifies retrospective cohort construction and supports translational research. Integration with computational tools, including natural language processing and image analysis, further enhances the ability to extract meaningful information from large datasets.

Preparation of research datasets requires careful attention to data governance. Digital systems can facilitate anonymization and structured data extraction, enabling the use of clinical material for research while maintaining patient privacy. These capabilities are particularly relevant for the development of artificial intelligence applications, which depend on large, well‐annotated datasets [10, 11].

Finally, integration of digital pathology with biobanking and molecular data resources supports a more comprehensive approach to biomedical research. By linking morphologic, clinical, and molecular information, digital platforms provide a foundation for data‐driven investigation and contribute to the evolving role of pathology in precision medicine [10, 11].

### 1.6 Clinical and Academic Impact of Digital Transformation

Beyond technical implementation, digital transformation has important implications for how pathology functions as a clinical and academic discipline [10, 12, 13]. By enabling real‐time access to diagnostic material across institutional boundaries, digital pathology reduces delays in consultation and facilitates more timely clinical decision‐making. This is particularly relevant in complex cases requiring subspecialty expertise, where rapid second opinions can directly influence patient management [4, 12, 13].

In academic settings, digital pathology also reshapes how knowledge is shared and how expertise is developed [10, 12, 13]. The ability to access large numbers of cases, including rare or complex diagnoses, allows trainees to learn in a more data‐rich environment than was possible with traditional glass slide collections. At the same time, pathologists can more easily collaborate across institutions, contributing to multi‐center studies and developing shared diagnostic standards [10, 12, 13].

From a strategic perspective, digital pathology may also influence how institutions position themselves within broader healthcare networks [4, 10]. Systems that successfully integrate workflow, data, and expertise are not only more efficient internally but may also function as regional or international hubs for diagnostic consultation, education, and research. In this sense, digital transformation extends beyond laboratory modernization and becomes a driver of institutional visibility and academic impact [4, 10, 13].

In practice, digital pathology has enabled more timely subspecialty consultation, expanded access to diverse case material for trainee education, and supported international diagnostic collaboration across institutional boundaries [10, 13].

### 1.7 Implementation Considerations and Practical Lessons

Successful digital transformation in pathology depends not only on technological adoption but also on practical implementation strategies that align with institutional workflows and organizational culture [5, 7, 10]. While the conceptual principles of digital pathology are broadly applicable, their translation into routine clinical practice requires careful attention to operational details.

One important consideration is the timing of implementation. Digital pathology systems are most effective when integrated into laboratory design from the outset, as demonstrated in the IUHW Narita model [5, 7]. In contrast, retrofitting digital systems into existing analog workflows often leads to hybrid processes that introduce inefficiencies and limit the potential benefits of digitization. Nonetheless, digital‐first principles are not limited to newly established institutions; existing laboratories can adopt these approaches through phased implementation and incremental workflow redesign, with expectations adjusted accordingly [4, 5, 7].

Another key factor is the alignment between laboratory processes and digital infrastructure. As described in earlier sections, scanner performance is closely linked to the rhythm of specimen processing. In practice, this means that laboratory staff, pathologists, and information technology teams must coordinate closely to ensure that slide production, digitization, and diagnostic review operate as a continuous system rather than as isolated steps [4, 5, 7].

User adoption also represents a critical determinant of success [5, 7]. Pathologists trained in microscope‐based workflows may initially experience challenges when transitioning to digital environments, particularly in relation to navigation, image interpretation, and workflow organization [5, 7, 13]. Structured training, gradual exposure, and the availability of hybrid diagnostic options during early implementation phases can facilitate this transition and improve acceptance. In addition, system design must account for variability in case types and diagnostic complexity. While routine cases can often be processed efficiently within standardized workflows, complex cases requiring extensive ancillary studies or multidisciplinary discussion may benefit from more flexible digital pathways. Designing systems that accommodate both high‐throughput routine work and complex diagnostic scenarios is therefore essential [5, 7, 10].

Finally, institutional governance and strategic planning play a central role in digital transformation [5, 10]. Decisions regarding infrastructure investment, data management policies, and network integration must be aligned with long‐term clinical and academic goals. In multi‐institutional settings, coordination across hospitals is necessary to ensure consistent standards, interoperability, and equitable distribution of diagnostic resources [4, 5, 10].

These practical considerations highlight that digital transformation is not a single technological upgrade but an ongoing organizational process [5, 10]. Institutions that approach digital pathology as a coordinated, system‐wide initiative are better positioned to develop sustainable and integrated diagnostic workflows. Successful adoption depends on clear governance structures, sustained institutional leadership, and realistic planning for long‐term operational costs [3, 4, 5]. Equally important are the human factors associated with implementation, including periods of hybrid workflow and increased cognitive load during early transition phases, which should be addressed through communication, training, and phased deployment. An important limitation of the IUHW Narita experience is the lack of direct pre‐ and post‐implementation quantitative comparisons. Unlike institutions that transitioned from established microscope‐based workflows, IUHW Narita Hospital was designed and opened as a digital‐first facility, without a preceding analog diagnostic workflow or legacy specimen‐processing system at the institution. Consequently, conventional before‐and‐after comparisons of turnaround time, workload distribution, user adoption, or operational costs cannot be performed within the same institutional setting. The workload data presented in Table 1 therefore illustrate the operational scale and sustainability of the system rather than demonstrating quantitative superiority over an analog workflow. Evaluation across institutions will therefore be necessary to determine the quantitative effects of digital‐first implementation on workflow and cost.

### 1.8 Challenges and Future Directions in Digital Pathology

Despite the rapid progress of digital pathology over the past decade, several challenges remain before fully integrated digital diagnostic environments become universal in clinical practice. These challenges involve not only technological infrastructure but also workflow adaptation, regulatory frameworks, and the evolving role of pathologists within increasingly data‐driven healthcare systems [3, 10, 13].

One of the most significant barriers to widespread adoption is the cost of digital pathology infrastructure. Whole‐slide scanners, high‐capacity storage systems, and high‐speed network infrastructure require substantial initial investment [3, 12]. In addition, long‐term data storage represents a continuing financial commitment, particularly as digital archives accumulate large volumes of whole‐slide image data over time. Digital pathology may also partially offset certain costs. Comprehensive digitization reduces dependence on large physical slide archives and the space required to store them, and it decreases expenses related to slide retrieval, duplication for consultation or teaching, and courier shipment for external review. Remote consultation can shorten turnaround times for second opinions while removing shipping and handling costs. These potential savings must be weighed against ongoing expenditures for data storage, network infrastructure, and IT support. Rather than assuming universal cost‑effectiveness, institutions should model their own cost structures and align digital pathology investment with broader strategic goals, such as access to subspecialty expertise and the creation of data resources for education and research [3, 4, 12].

Another important challenge involves workflow redesign and organizational change [3, 5, 7]. Pathology laboratories have historically developed highly optimized processes centered around microscope‐based diagnosis. Transitioning to a digital‐first model requires adjustments not only in laboratory operations but also in diagnostic habits and professional culture. Pathologists must become comfortable navigating digital slides, laboratory staff must adapt specimen processing workflows to support scanning pipelines, and information technology teams must integrate digital pathology systems with existing hospital infrastructure [5, 7, 10].

Regulatory considerations also play a role in shaping digital pathology implementation. In many countries, whole‐slide imaging systems must undergo validation for diagnostic use, with requirements related to image quality, workflow integration, and documentation of equivalency with conventional microscopy. Regulatory expectations are also expanding for artificial intelligence tools, including clinical validation, transparency regarding training data, and ongoing performance monitoring. In Japan, the Japanese Society of Pathology has published guidance on the use of artificial intelligence in pathological diagnosis, addressing issues such as validation, quality assurance, and clinical responsibility [5, 14, 15]. Telepathology across institutional or national boundaries introduces additional considerations related to licensing, data security, and patient privacy [4, 5, 9]. In the IUHW network, international collaboration has been structured as a formal consultation model, in which cases are reviewed by local pathologists and remote input is provided as a second opinion. This approach, currently implemented with a partner institution in Vietnam and under development for Mongolia, is designed to accommodate licensing and jurisdictional constraints by maintaining primary diagnostic responsibility within the local institution, and may provide a practical framework for institutions developing international telepathology programs. As digital pathology networks expand, harmonization of regulatory standards will become increasingly important [4, 5, 9].

Another emerging area of attention involves data governance and cybersecurity. Digital pathology systems store large volumes of clinically sensitive information, including high‐resolution tissue images and associated patient data [5, 8]. Ensuring secure storage, controlled access, and compliant data sharing is essential, particularly when digital slides are used for research collaborations or artificial intelligence development [10, 11, 13].

The integration of computational tools into pathology workflows presents both opportunities and challenges [10, 11, 13]. Advances in artificial intelligence and image analysis have generated significant enthusiasm for computational pathology applications, ranging from automated tumor detection to quantitative biomarker analysis [10, 11, 14]. However, translating these tools into routine clinical practice requires careful validation, regulatory approval, and thoughtful integration into existing diagnostic workflows [10, 11, 14]. AI systems are most likely to succeed when they function as decision‐support tools that assist pathologists with repetitive or quantitative tasks while preserving the central role of human expertise in diagnostic interpretation [10, 13, 14, 16].

As digital pathology increasingly supports artificial intelligence and cross‐institutional data sharing, ethical and governance considerations will become increasingly important. Issues related to privacy, secondary use of clinical material, dataset bias, explainability, and trust should be incorporated into system design from early stages [10, 11, 14]. Furthermore, the applicability of digital pathology in diverse healthcare environments, including resource‐limited settings, highlights both its potential to expand access to subspecialty expertise and the infrastructural and regulatory challenges that remain [9, 10].

Looking ahead, the future of digital pathology will likely involve increasing integration with other forms of medical data. Pathology images, radiologic studies, genomic profiles, and clinical information can potentially be analyzed together within unified diagnostic platforms [10, 11, 13]. Such multimodal integration may help reveal new relationships between tissue morphology and molecular biology, contributing to more precise disease classification and personalized treatment strategies.

As these developments unfold, the role of the pathologist will continue to evolve. Rather than being replaced by automation, pathologists will increasingly function as interpreters and integrators of complex diagnostic data [10, 13, 14, 16]. Digital pathology platforms provide tools for visualizing and analyzing large datasets, but the clinical meaning of these findings must still be interpreted within the biological and clinical context of each patient. In this sense, digital transformation expands rather than diminishes the intellectual scope of pathology practice.

Ultimately, the most successful digital pathology systems will be those that support the fundamental goals of pathology: accurate diagnosis, effective communication with clinicians, high‐quality education of future physicians, and meaningful contributions to biomedical research [10, 13, 14, 16, 17]. Achieving these goals requires thoughtful integration of technology with the professional expertise and judgment that remain central to the discipline.

### 1.9 Generalizable Principles of Digital Transformation in Pathology

The experience described in this review suggests that digital transformation in pathology is not defined by the adoption of specific technologies, but by a set of underlying design principles that can be applied across different institutional contexts. Large‐scale digital pathology implementations have evolved through different institutional priorities and operational contexts, as summarized in Table 2. Studies from Memorial Sloan Kettering and several UK centers have focused mainly on validation, diagnostic concordance, and efficiency, whereas the Utrecht experience described the transition of an academic laboratory to fully digital practice [18, 19, 20]. Other reports have addressed the financial implications of implementation or the use of digital pathology across hospital networks [20, 21, 22]. IUHW Narita shares many features with these programs, but was designed from the outset to connect continuous‐flow laboratory processing, routine digital diagnosis, and networked pathology within the same system. These experiences indicate that implementation should be adapted to the needs and structure of each institution rather than follow a single model.

**Table 2:** Examples of large‐scale digital pathology implementation models and their distinguishing features.

Institution/model	Region	Setting	Implementation emphasis	Distinguishing features
IUHW Narita model	Japan	Multi‐site academic and hospital network	Workflow‐driven, digital‐first, network‐integrated design	Continuous‐flow laboratory process; comprehensive digitization; domestic and international consultation network
Memorial Sloan Kettering Cancer Center	USA	Large academic cancer center	Large‐scale WSI validation and clinical workflow evaluation	Large‐scale WSI validation; efficiency and concordance assessment [18]
Utrecht University Medical Center	Netherlands	Academic pathology laboratory	Fully digital diagnostic practice	Institution‐wide digital workflow; strong emphasis on change management and staff adaptation [19]
UK breast pathology implementation	UK	Multi‐center breast pathology service	Validation and implementation for primary diagnosis	Screen‐detected breast lesions; concordance analysis and practical experience from four centres [20]
Kaiser Permanente	USA	Integrated health care organization	System‐wide business‐case‐driven implementation	Emphasis on scalability, operational planning, and cost modeling [21]
Spanish multi‐center network	Spain	Multi‐hospital network	Centralized digital platform with distributed diagnosis	Shared infrastructure across hospitals; multi‐site consultation and standardization [22]

First, digital pathology should be approached as a workflow‐driven process rather than a technology‐driven upgrade [5, 6, 7]. The efficiency and scalability of digital systems depend on the alignment of pre‐analytical laboratory processes with the requirements of continuous slide digitization. Without such alignment, even advanced imaging systems may fail to improve overall performance [5, 7].

Second, digital transformation requires the establishment of a digital‐first diagnostic environment. Selective or partial digitization often leads to hybrid workflows that introduce inefficiencies and limit the integration of digital data into routine practice [3, 5, 13]. In contrast, comprehensive digitization enables consistent access to diagnostic material and supports the development of integrated clinical, educational, and research workflows [3, 6, 13].

Third, data integration is essential. Whole‐slide images should not be treated as isolated visual artifacts, but as components of a broader information ecosystem that includes laboratory data, clinical records, and molecular results [2, 6, 10, 13]. Systems that facilitate seamless access to these data sources enhance diagnostic context and enable more informed clinical decision‐making [2, 6, 13].

Fourth, interoperability and system flexibility are critical for long‐term sustainability. The use of standardized formats and vendor‐neutral architectures reduces the risk of technological lock‐in and allows institutions to adapt their infrastructure as technologies evolve.

Finally, digital pathology enables the transition from institution‐centered to network‐based diagnostic models. By supporting remote access and shared workflows, digital systems allow expertise and resources to be distributed across multiple sites, improving access to specialized diagnostics and enabling new forms of collaboration [4, 6, 9, 13].

These principles are not unique to IUHW Narita and are also evident in other digital pathology programs [4, 5, 6, 7, 13, 18, 19, 20, 21, 22]. The IUHW Narita model represents one way of combining them across laboratory workflow, diagnosis, data management, and networked practice. Their application will necessarily differ among institutions according to local needs and resources. These principles are summarized in Table 3.

**Table 3:** Generalizable principles of digital transformation in pathology.

Principle	Definition	Practical implication
Workflow‐driven design	Digital pathology begins before scanning	Redesign processing, staining, tracking
Digital‐first diagnosis	Digital slides become primary diagnostic material	Avoid selective/hybrid workflows
Data integration	Whole‐slide images are part of clinical data ecosystem	Link laboratory information system, clinical data, prior cases
Interoperability	Avoid vendor lock‐in	Use standardized formats such as DICOM
Networked diagnostics	Expertise can be distributed	Enable subspecialty consultation and multi‐site collaboration

## 2 Conclusion

The transition from analog microscopy to digital pathology represents a fundamental shift in how pathology is practiced [6, 13]. While early efforts in digital pathology primarily focused on slide digitization, true digital transformation requires a coordinated redesign of laboratory workflows, diagnostic environments, and information infrastructure [5, 6]. As illustrated by the experience at IUHW Narita Hospital, a digital‐first approach can integrate these elements into routine clinical practice and support scalable diagnostic operations.

A key insight from this transformation is that digital pathology should not be viewed merely as a technological upgrade, but as a systems‐level reconfiguration of pathology services [5, 6]. Workflow optimization, data integration, and network connectivity are interdependent components that must be aligned to fully realize the benefits of digital systems [5, 6, 8]. Without such alignment, the introduction of digital tools alone may result in hybrid workflows that limit efficiency and fail to leverage the full potential of whole‐slide imaging [3, 5].

Beyond improvements in laboratory efficiency, digital pathology enables new models of collaboration, education, and research [4, 10, 13]. Networked diagnostic systems expand access to subspecialty expertise across institutional boundaries, while digital archives enhance diagnostic safety and provide scalable platforms for training [4, 10]. In addition, the integration of whole‐slide images with clinical and molecular data supports the development of data‐driven approaches to disease classification and management [10, 13].

Looking forward, digital pathology is likely to become a foundational platform for precision medicine [10, 11, 14]. The integration of morphologic, radiologic, and genomic data within unified diagnostic environments may enable more comprehensive understanding of disease processes and support the development of personalized therapeutic strategies [10, 11]. At the same time, challenges related to cost, data governance, regulatory frameworks, and workflow adaptation must be addressed to ensure sustainable implementation [3, 5, 12].

In conclusion, digital transformation in pathology represents a shift from image‐based diagnosis toward data‐integrated and networked diagnostic systems [6, 13]. The IUHW Narita model is one example of this transition, and comparison with other large‐scale implementations shows that the optimal approach depends on the setting and needs of each institution [18, 19, 20, 21, 22]. For institutions moving beyond partial digitization, workflow design, digital‐first diagnosis, interoperability, and networked practice need to be considered together. Integration of these components will be important for realizing the broader clinical and academic potential of digital pathology.

## Author Contributions

Hiroyuki Kusano and Takayuki Shiomi conceptualized and wrote the manuscript.

## Conflicts of Interest

The authors declare no conflicts of interest.

## Data Availability

Data sharing not applicable to this article as no datasets were generated or analysed during the current study.
